# Lifestyle and precision diabetes medicine: will genomics help optimise the prediction, prevention and treatment of type 2 diabetes through lifestyle therapy?

**DOI:** 10.1007/s00125-017-4207-5

**Published:** 2017-01-25

**Authors:** Paul W Franks, Alaitz Poveda

**Affiliations:** 1 Department of Clinical Sciences, Genetic and Molecular Epidemiology Unit, Clinical Research Centre, Lund University, Jan Waldenströms gata 35, Skåne University Hospital, Malmö, SE-20502 Sweden; 2 Department of Public Health and Clinical Medicine, Section for Medicine, Umeå University, Umeå, Sweden; 3 Department of Nutrition, Harvard T.H. Chan School of Public Health, Boston, MA USA

**Keywords:** Biomarkers, Lifestyle, Precision medicine, Review, Type 2 diabetes

## Abstract

Precision diabetes medicine, the optimisation of therapy using patient-level biomarker data, has stimulated enormous interest throughout society as it provides hope of more effective, less costly and safer ways of preventing, treating, and perhaps even curing the disease. While precision diabetes medicine is often framed in the context of pharmacotherapy, using biomarkers to personalise lifestyle recommendations, intended to lower type 2 diabetes risk or to slow progression, is also conceivable. There are at least four ways in which this might work: (1) by helping to predict a person’s susceptibility to adverse lifestyle exposures; (2) by facilitating the stratification of type 2 diabetes into subclasses, some of which may be prevented or treated optimally with specific lifestyle interventions; (3) by aiding the discovery of prognostic biomarkers that help guide timing and intensity of lifestyle interventions; (4) by predicting treatment response. In this review we overview the rationale for precision diabetes medicine, specifically as it relates to lifestyle; we also scrutinise existing evidence, discuss the barriers germane to research in this field and consider how this work is likely to proceed.

**Electronic supplementary material:**

The online version of this article (doi:10.1007/s00125-017-4207-5) contains a slideset of the figures for download, which is available to authorised users.

## Introduction

The major developments in genomic technologies and their application to large, well characterised collections of samples have led to the generation of extensive new knowledge about disease biology. This has inspired new avenues for type 2 diabetes prevention, treatment and cure that are inherent to the concept of precision medicine. According to the National Research Council [1], precision medicine is not intended to involve the complete personalisation of medical devices and therapies; instead, it should focus on the classification of ‘individuals into subpopulations that differ in their susceptibility to a particular disease, in the biology and/or prognosis of those diseases they may develop, or in their response to a specific treatment’ with the expectation that ‘preventive or therapeutic interventions can then be concentrated on those who will benefit, sparing expense and side effects for those who will not’. By this and most other definitions, precision medicine focuses on applying biomarker technologies to the individual patient to help improve prediction and assessment of: (1) risk-factor susceptibility; (2) disease stratification; (3) prognosis; and (4) treatment response.

Our understanding of type 2 diabetes pathobiology has improved dramatically recently, owing largely to a quantum leap in human genome sequencing, precipitated by ground-breaking achievements made in the preceding century, including mapping of the *Drosophila* genome [2] and the structural characterisation of DNA [3]. These were the foundations for human genome sequencing [4, 5] and affordable technologies for high-resolution characterisation of the genome, metagenome, epigenome, transcriptome, proteome, and metabolome. Combined with novel bioinformatics and the emergence of large global collaborative networks, exciting possibilities have emerged for the prediction and prevention of disease in ways that are more personal and precise than ever before.

**Fig. 1:**
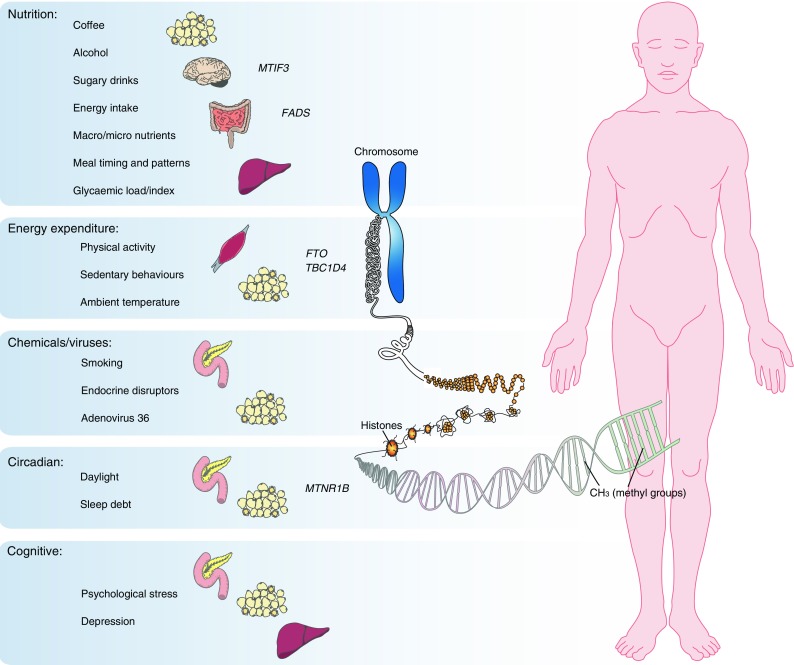
Type 2 diabetes results from the complex interplay between environmental and genomic factors. The model is thus one of primers and catalysts, whereby environmental triggers act against a backdrop of genetic susceptibility to affect the transcriptional and regulatory processes that cause diabetes (e.g. through methylation, chromatin remodelling or histone modifications). The figure shows the key lifestyle risk factors, candidate loci (with evidence of gene–lifestyle interactions) and target organs purported to affect adiposity and/or glycaemic control

Genetic variation is quite literally the starting point of the biological cascade that underpins phenotypic expression (known as the ‘central dogma of molecular biology’) [6]. But for complex diseases like type 2 diabetes, genetics is by no means the absolute determinant, and thus an enormous amount of downstream work remains before we will adequately understand how genetic and lifestyle factors (e.g. nutrition, exercise, medications and stress) work jointly to affect gene transcription and translation, and phenotypic expression (Fig. 1). The model is thus one of primers and catalysts: environmental triggers in the context of genetic predisposition.

Given that significant changes in human genetic variation manifest over many generations, the consensus is that the global surge in type 2 diabetes prevalence is caused predominantly by the rapid and widespread adoption of obesogenic lifestyles. Metabolic dyshomeostasis is a common consequence of unhealthy lifestyles, driven by disturbed substrate production and/or metabolism in the liver, skeletal muscle and adipose tissue, or by interfering with the synthesis, secretion or action of insulin. However, diabetes pathophysiology is complex and heterogeneous with multiple feedback loops, such that people vary in susceptibility to risk factors and response to therapies, and the molecular defects that cause the disease in a given patient are rarely known. Nevertheless, the measurement or prediction of primordial factors might facilitate more effective diabetes prevention if they helped determine the specific risk factors to which a person is susceptible and the therapies they are likely to respond well to.

## Lifestyle in type 2 diabetes

In most people at risk of or with type 2 diabetes, prognosis can be improved by enhancing peripheral insulin sensitivity. Although oral glucose-lowering agents are often used for this purpose, reducing energy intake and increasing non-resting energy expenditure (physical activity) are highly effective first line therapeutic options, with associated reduced lipid content in or around adipose, liver and muscle tissue being pivotal to this process.

Although reduction of energy intake and an increase in physical activity can both cause weight loss, they do so through contrasting states of low and high metabolic turnover respectively, which involve the activation of different molecular pathways and processes that encompass both common and unique corresponding health benefits. Accordingly, some patients will respond well and others poorly to the same intervention, with response being determined in part by individual biology, but also by psychosocial factors that influence adherence and perceived success.

### Non-resting energy expenditure

Non-resting energy expenditure constitutes activities that are either structured, with the intent of improving fitness or sports skills (i.e. exercise training), or activities that are not explicitly intended as exercise (e.g. active commuting, gardening, dog walking), as well as subconscious movements (e.g. fidgeting). Regardless of mode, regular physical activity can improve glucose homeostasis through insulin-dependent mechanisms (e.g. by improving the sensitivity of peripheral tissue to insulin) and insulin-independent mechanisms (e.g. muscle contraction, shear stress, reductions in hepatic glucose production), thereby reducing pancreatic beta cell stress, and helping to prevent or slow progression of diabetes.

### Diet

Diet also plays many complex roles in metabolic homeostasis. Frequently, the perceived link between diet and type 2 diabetes is through excess energy intake that leads to obesity, and over-consumption of refined carbohydrates that rapidly raises blood glucose and places a compensatory demand on the beta cells for endogenous insulin. However, there are many other roles diet plays in affecting diabetes risk: zinc, for example, regulates insulin storage in the secretory granules of the pancreatic beta cells, and functional variants within *SLC30A8,* encoding a zinc transporter, affect this process [7]; and long chain polyunsaturated fatty acids are ligands for fatty acid receptors, like peroxisome proliferator-activated receptor gamma (PPARγ*)* [8]. Overall diet quality (e.g. Mediterranean diet) is also an important feature of many successful diabetes prevention programmes [9].

### Body composition

Adequately characterising lifestyle exposures is thus important, but so too is how body corpulence is defined. Emphasis is often on total adiposity (e.g. weight change); however, the regional distribution of adipose tissue (particularly when it is deep within the abdominal cavity and within or around the liver, heart and pancreas) [10] and the patterns of change in response to an intervention [11], are likely to elucidate diabetes aetiology better than weight change per se. The emergence of optical triangulation 3D scanning technologies that allow frequent assessments of body form to be captured quickly, safely and at relatively low cost, may facilitate this process [12].

Implementing structured lifestyle interventions for diabetes prevention in clinical practice is cost effective [13]. Behavioural assessment is a critical part of this process, helping to identify key risk factors, determine appropriate intervention targets and gauge adherence. Ideally such assessments would be performed through the diabetes care system, but time and cost limitations are major barriers to the implementation of lifestyle medicine and careful lifestyle assessments are rarely undertaken in practice [14]. This is unfortunate, as appropriate lifestyle monitoring and tailoring of lifestyle advice may help further improve the efficacy and cost effectiveness of lifestyle medicine.

## How precision lifestyle medicine might work

**Fig. 2:**
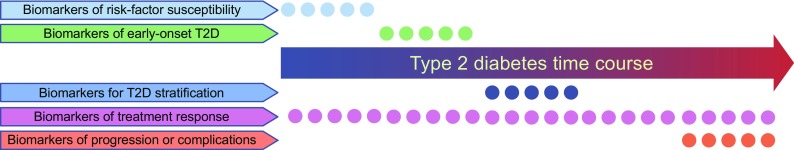
Precision medicine for type 2 diabetes. A schematic showing key time points for intervention in the course of type 2 diabetes (T2D) pathophysiology where precision lifestyle medicine might play a role


Precision medicine in type 2 diabetes is very much at a theoretical stage, particularly as it relates to personalised lifestyle therapy. However, its successes in other diseases and with pharmacotherapy offer a glimpse of how tailored lifestyle advice for type 2 diabetes prevention, guided by personal genomic data, might be achievable (Fig. 2 and text box: Precision lifestyle medicine in type 2 diabetes):

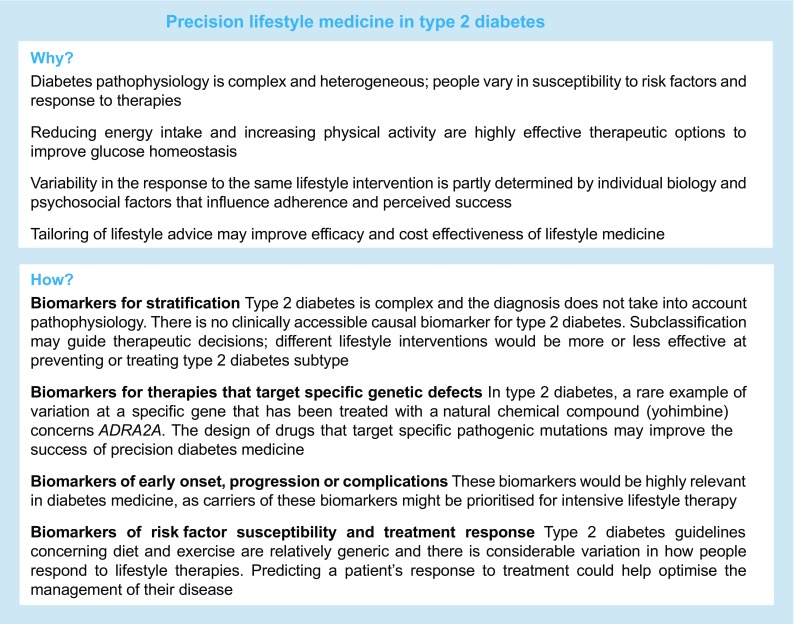


### Biomarkers for type 2 diabetes stratification

An excellent example of precision diabetes medicine can be found in MODY. The disease, often misdiagnosed as type 1 diabetes owing to the young age of onset and insulin deficiency, can be accurately and precisely diagnosed using genetic screening, leading to subclassification and highly effective targeted drug therapy.

From an aetiological perspective, type 2 diabetes is far more complex than MODY, and in type 2 diabetes the direct diagnostic assay is restricted to blood glucose (or glycosylated haemoglobin). However, elevations in blood glucose are usually the consequence of other cellular defects in the liver, pancreas, skeletal muscle and other peripheral tissues, primarily affecting the endogenous production of glucose and insulin and the rate of glucose metabolism. There is no clinically accessible causal biomarker for type 2 diabetes, although markers of beta cell function are often used to help diagnose other causes of abnormal blood glucose concentrations. Thus, diagnosing type 2 diabetes and distinguishing it from other causes of elevated blood glucose is challenging and rarely elucidates the primary cause of the disease.

Subclassification of type 2 diabetes using biomarkers and other information might not pinpoint specific molecular causes, but it may bring the diagnosis closer to that point and guide therapeutic decisions. In a recent study conducted in the USA, the use of electronic medical records for the classification of type 2 diabetes into subtypes (coinciding with cardiovascular diseases, neurological diseases, allergies and HIV infections) that were subsequently genetically characterised provides an example of how type 2 diabetes diagnoses might be refined through genotype-guided patient stratification [15]. Moreover, amongst the millions of people diagnosed with type 2 diabetes annually undoubtedly reside those with rare monogenetic disorders that appear like type 2 diabetes, yet are caused by rare single gene mutations. These mutations might be targetable with specific treatments, such as recently described for the *SUR1* (also known as *ABCC8*) locus [16]. Conceivably, different lifestyle interventions, particularly those with a nutritional emphasis, would be more or less effective at preventing or treating each type 2 diabetes subtype, thus providing avenues for personalised lifestyle therapy.

### Biomarkers for therapies that target specific genetic defects

Some of the most remarkable successes in precision medicine to date have involved the design of drugs that target specific pathogenic mutations. Two striking examples are drugs for treating chronic myelogenous leukaemia and lung adenocarcinoma, imatinib (Glivec/Gleevec) and crizotinib (Xalkori), respectively. Imatinib targets the protein product of a novel fusion gene, *BCR*–*ABL* [17], whereas crizotinib targets a genetic abnormality (a fusion gene called *EML4–ALK*) caused by the inversion of the anaplastic lymphoma kinase (*ALK*) gene [18]. In type 2 diabetes, a rare example of a specific gene defect that has been successfully treated with naturally occurring chemical compounds is that of the α2A adrenergic-receptor (α_2A_AR) encoding gene, *ADRA2A* [19]. Here, an *ADRA2A* variant causes type 2 diabetes as a result of impaired insulin secretion owing to receptor overexpression; treatment with the naturally occurring indole alkaloid, yohimbine (a chemical compound extracted from *Pausinystalia johimbe* tree bark) blocks the receptor and improves insulin secretion.

### Biomarkers of early-onset, progression or complications

Biomarkers of the early-onset or progression of type 2 diabetes, or of the onset of diabetes-associated complications, would be highly relevant for diabetes therapy. Carriers of these biomarkers might be prioritised for intensive lifestyle therapy before or soon after the disease is manifest, much as people with a family history of diabetes are treated today. However, no tangible examples of these biomarkers currently exist.

### Biomarkers of risk-factor susceptibility and treatment response

Type 2 diabetes guidelines concerning diet and exercise are relatively generic. Such guidelines are typically derived from epidemiological studies on the risk attributable to modifiable lifestyle exposures and clinical trials showing that intervening with those same factors reduces risk. Importantly, these data reflect population averages, often with wide confidence intervals, indicating that, although targeting risk factors diminishes type 2 diabetes risk on average, there are people within intervention groups who improve greatly (so called ‘super-responders’), and others who may not improve at all (so called ‘non-responders’) or whose condition worsens (so called ‘adverse responders’). One of the first studies on blood glucose variability in response to chronic exercise showed that although mean insulin sensitivity increased by 10% following the intervention 42% of the participants experienced no change or became more insulin resistant [20]. According to a recent review, the proportion of non-responders to exercise training regarding glucose homeostasis ranged between 7% and 63% [21] and the number of adverse responders averaged 8.4% [22]. The relatively high proportion of people who do not appear to respond well to exercise has motivated the search for the underlying mechanisms [23], with the assumption that genetic and epigenomic variation play a key role. However, as we discuss later, how much of this variability is of biological origin and how much is driven by other factors remains unclear.

## Evidence base: strengths and weakness

Early twin and family studies showed that response to diet and exercise interventions vary to a significantly greater extent between sibships and pedigrees than within them, suggesting a heritable component to some treatment response phenotypes [24–26]. A 100-day overfeeding protocol conducted in 12 pairs of monozygotic (MZ) male twins showed, for example, that the gain in fat mass was roughly three times more similar within the MZ twin pairs than between non-twins [26]. Elsewhere, a study conducted to investigate the response to a negative energy balance in seven pairs of MZ male twins found that the variance in change in fat mass was 14.1 times more similar within than between twin pairs [25].

When used to model interactions between common genetic variants and lifestyle exposures, data from observational studies can generate hypotheses relevant to treatment response and risk-factor susceptibility. However, the inherent limitations of epidemiology (chance, bias, confounding), the difficulties in accurately and precisely assessing phenotypes and lifestyle behaviours in free-living populations, as well as challenges that specifically hinder the estimation of interaction effects in observational data (e.g. heteroscedasticity and scale dependency) necessitate caution when interpreting the causal relevance of observational data. In interaction studies, the imperative of replication has been especially difficult to achieve owing to winner’s curse, heterogeneous study designs, environmental idiosyncrasies, etc. (see [27]). Nevertheless, a complete absence of replication in the face of concerted, adequately powered attempts undermines the value of the original findings, as they likely reflect idiosyncratic effects or false-positives.

There is little extensively replicated epidemiological evidence of gene–lifestyle interactions, with the exception of those at the *FTO* locus (see text box: What we know about the *FTO*–physical activity interaction). Soon after the finding that *FTO* variation affects obesity risk, data emerged from two epidemiological studies showing that *FTO* variation may modify the relationship between physical activity and estimates of adiposity in European [28, 29] and North American [30] adults. An analysis of clinical trial data from the Diabetes Prevention Program (DPP) found no evidence that *FTO* modified the effects of lifestyle intervention on weight loss, although there was weak evidence that an interaction might affect subcutaneous adipose mass [31]. Many studies followed, but with mixed results; therefore, we undertook a large meta-analysis (*N* = 220,000) to test the hypothesis, which confirmed the presence of an interaction [32]. Those findings were recently extended in the UK Biobank [33], where statistically robust interaction effects were reported between the *FTO* variant and physical activity, frequency of alcohol consumption, sleep duration, diet and salt consumption. Cross-sectional analyses focusing on lifestyle interactions with genetic risk scores comprised of other obesity-associated loci have also been widely replicated, although the extent to which those interactions are driven by *FTO* is rarely described.
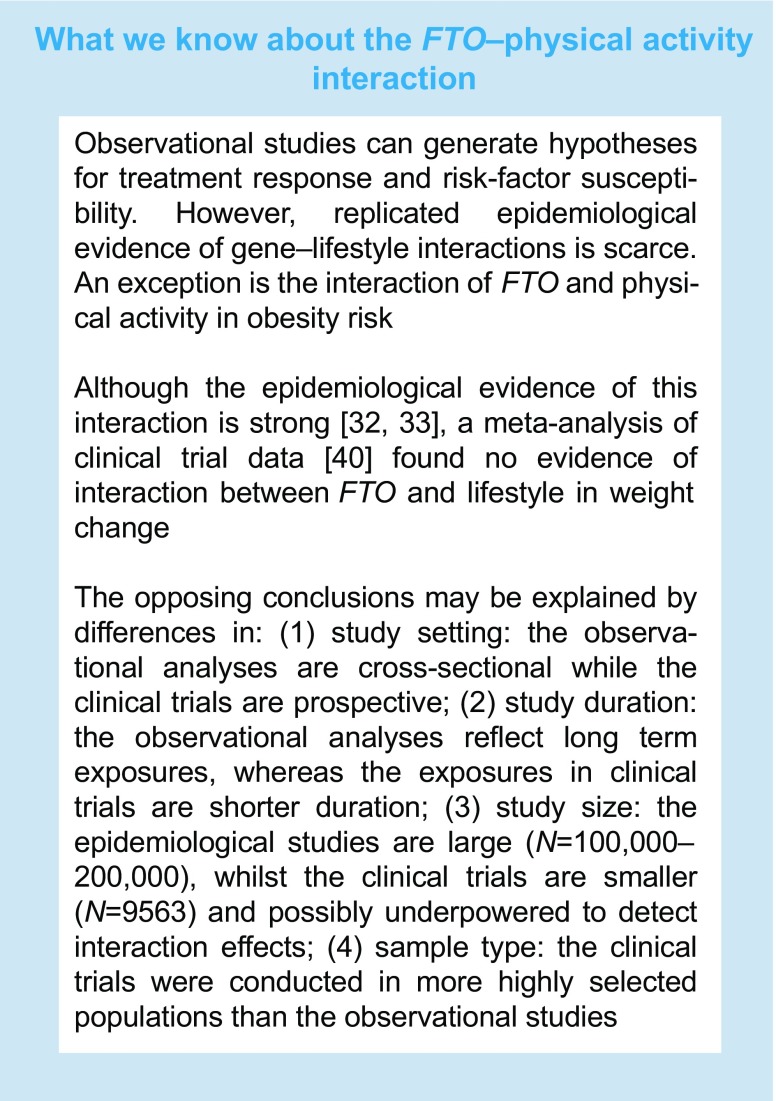


While the epidemiological data seem compelling, it is predominantly cross-sectional and evidence of gene–lifestyle interactions in large observational datasets may be biased or confounded. Hence, epidemiological evidence of gene–lifestyle interactions should not only be replicated but also supported by other types of causal evidence before exploiting the findings for the purposes of genotype-guided lifestyle prescription. There are some functional data positioning *FTO* as a plausible candidate for lifestyle interactions (e.g. *FTO* encodes a 2-oxoglutarate oxygenase that is highly expressed in hypothalamic nuclei in mice and humans [34], and phosphocreatine and inorganic phosphate levels have been shown to recover more rapidly following exhaustive exercise in *FTO* (rs9939609) risk allele carriers [35]), but the discovery of long-range activation of *IRX3* and *IRX5* by *FTO* [36, 37] suggests that simple interpretations about how *FTO* and lifestyle interact are unlikely to be accurate.

Nevertheless, while understanding the functional basis of interactions is desirable, the absence of this knowledge does not preclude using evidence of gene–lifestyle interactions for precision medicine, whereas demonstrating cause and effect in trials is essential. A recent systematic review of selected published trial data concluded that *FTO* variants modify weight loss in response to lifestyle interventions [38]. However, the meta-analysis of published data for gene–lifestyle interactions may be biased [39], and a subsequent meta-analysis of clinical trial data, which adopted a more rigorous and more inclusive approach involving de novo, standardised interaction analyses, found no evidence of interaction between *FTO* and lifestyle [40]. A recent clinical trial analysis (DPP and Look AHEAD [The Action for Health in Diabetes] studies) that focused on the spectrum of BMI-associated variants reached similar conclusions for *FTO* and almost all other variants assessed; the exception was for *MTIF3*, which showed evidence of gene–lifestyle interactions in the trials [41], as well as in epidemiological cohorts [42].

**Fig. 3:**
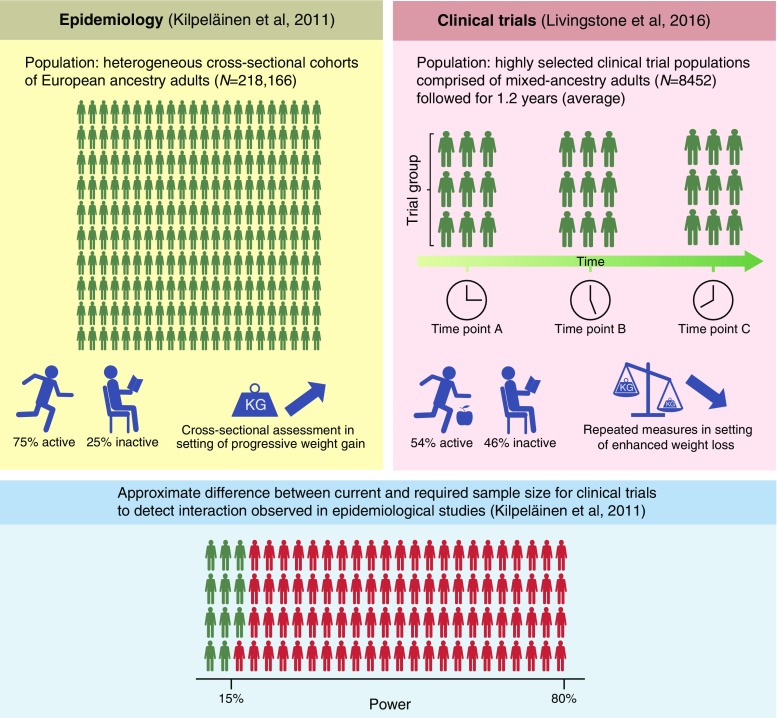
Estimating the required power for clinical trials focused on interaction effects of diabetes risk factors that have been previously reported in epidemiological studies. The figure compares two core studies focused on the interaction of *FTO* variants and lifestyle in obesity. We sought to estimate the power that the sample size reported by Livingstone et al [40] had to detect the interaction of the *FTO* variant and physical activity in obesity, as previously described in Kilpeläinen et al [32]. The conclusions regarding power and sample size in trials in this figure are predicated on the assumption that the interaction effect reported in the cross-sectional epidemiological analysis [32] can be applied to the setting of a randomised lifestyle intervention meta-analysis. However, there are several factors that are likely to confound this comparison; these are outlined in the figure. The given estimates of power and sample size are intended only to illustrate that the trials are likely to be substantially underpowered to observe previously reported interaction effects, rather than provide precise estimates of these variables

Although the starkly opposing conclusions drawn about *FTO* from the observational and clinical trial data appear contradictory, they are potentially reconcilable. For example, the observational analyses [32] are cross-sectional and may reflect the modifying effects of very long-term lifestyle exposures and outcomes, whereas the clinical trials [40] are prospective and confined to a relatively short intervention period (ranging from 8 weeks to 3 years). Moreover, the epidemiological studies are large (*N* = 100,000–200,000) and apparently adequately powered, whereas the trials analysis is an order of magnitude smaller (*N* = 9563), may have been underpowered to detect interaction effects, and was conducted in very selected populations compared with the observational studies (Fig. 3). Nevertheless, the absence of an interaction effect in the trials indicates that, for the benefit of enhancing weight loss for diabetes prevention, there may be little clinical value in tailoring common lifestyle interventions to *FTO* genotype.

## The future

Determining the causal effects of lifestyle, even in randomised controlled trials, is much more challenging than for drug therapies, not least because most lifestyle interventions cannot be masked and no placebo exists. Thus, a participant’s behaviours during a lifestyle trial that affect the trial’s outcomes may change as a consequence of the intervention in ways that are not expected or measured. This phenomenon was first described in a study of older men and women (58–78 years old) who underwent an 8 week supervised aerobic training programme (cycling three times per week) [43]. Objective assessments of total energy expenditure were made using doubly labelled water, resting metabolic rate by respiratory gas exchange, and physical activity energy expenditure using the latter values combined with information about sleep and exercise. The authors found that despite an increase in exercise energy expenditure of 628 kJ/day (150 kcal/day), non-exercise-activity energy expenditure decreased proportionately and no overall change in total energy expenditure was hence observed.

When one considers that exercise interventions deployed in clinical trials typically occupy about 150 mins per week, or about 1.5% of the total time, it is possible that compensatory behaviours underlie much of the apparent heterogeneity in response. Even in tightly controlled inpatient studies, unintended variations in the participants’ behaviours can hinder data interpretation. For example, in one of the studies cited above, 84 days of overfeeding with restricted physical activity resulted in an average weight gain of 8.1 kg (range: 4.3–13.3 kg) [26]. The very lowest and highest weight gains suggest that the diet and exercise regime was not strictly followed, as a weight gain of 4.3 kg with this level of overfeeding accounts for only ∼40% of the predicted weight gain for that participant and would require energy expenditure equivalent to running ∼17–29 km each week during the intervention. By contrast, a weight gain of 13.3 kg would require complete indolence during the protocol. While individual biological variation in energy metabolism might explain some of these differences in weight gain, it seems probable that lack of adherence to the study protocol is the predominant explanation.

In current research and practice, lifestyle behaviours are frequently assessed through self-report methods, despite some key limitations [44]. However, numerous wearable technologies exist that are relatively inexpensive, suitable for long-term monitoring, and valid, albeit also with important caveats [45]. Nevertheless, modern wearables, used in combination with self-report methods have tremendous potential to characterise health-related behaviours and exposures, as the continuous assessment of movement, sleep, temperature, blood glucose and other relevant factors is possible without apparently impacting behaviour [46]. The use of such devices in epidemiology and clinical trials is likely to be necessary to delineate biological drivers of risk-factor susceptibility and treatment response from other sources of error and bias that cause heterogeneity. The continuous assessment of quantitative phenotypes germane to a clinical trial’s outcomes would also help overcome a further major limitation of some lifestyle trials that only assessed outcomes at enrolment and the trial’s end. In those settings, regression dilution, which occurs when changes in quantitative traits are inferred from too few data points, generates error. Thus, continuous (or frequently repeated) assessments of the trial’s quantitative outcomes would further help isolate response variability from error. In a recent eloquent study that focused on personalised diets, Zeevi and colleagues collected detailed diet data and objectively assessed physical activity and blood glucose in 800 Israeli adults during a 1 week observational phase. In combination with gut microbiota data, the authors were able to predict individual postprandial glucose excursions to specific foods and designed personalised diet interventions that improved glucose control [47]. This approach had the added benefit of allowing the background heterogeneity in the participants’ lifestyles to be factored into the intervention design, further reducing error.

## Summary

Although evidence of specific genetic loci that modulate risk-factor susceptibility and treatment response in type 2 diabetes is weak, the rationale, which is supported by data from twin and family studies, remains strong, motivating continued research in this area. For example, the National Institutes of Health’s (NIH’s) Common Fund initiative called the Molecular Transducers of Physical Activity (http://commonfund.nih.gov/MolecularTransducers), is a multimillion dollar funding programme focused on defining ‘optimal physical activity recommendations for people at various stages of life’ and developing ‘precisely targeted regimens for individuals with particular health needs.’ Nevertheless, this topic is clearly challenging, and to realise the vision of the NIH and others will likely require new study designs and analytical methods that overcome the major barriers to precision lifestyle medicine in type 2 diabetes.

## Electronic supplementary material

Below is the link to the electronic supplementary material.

ESM Downloadable slideset (PPTX 410 kb)

## Abbreviations

DPP: Diabetes Prevention Program
MZ: Monozygotic
NIH: National Institutes of Health
