# Supplementary material for: Lifestyle and precision diabetes medicine: will genomics help optimise the prediction, prevention and treatment of type 2 diabetes through lifestyle therapy?
Source: Diabetologia. 2017 Jan 25;60(5):784–92. doi: 10.1007/s00125-017-4207-5 (PMC6518113; doi:10.1007/s00125-017-4207-5)
Supplement: Supplementary file 1 — Downloadable slideset (PPTX 410 kb) [file 125_2017_4207_MOESM1_ESM.pptx]

## Slide 1
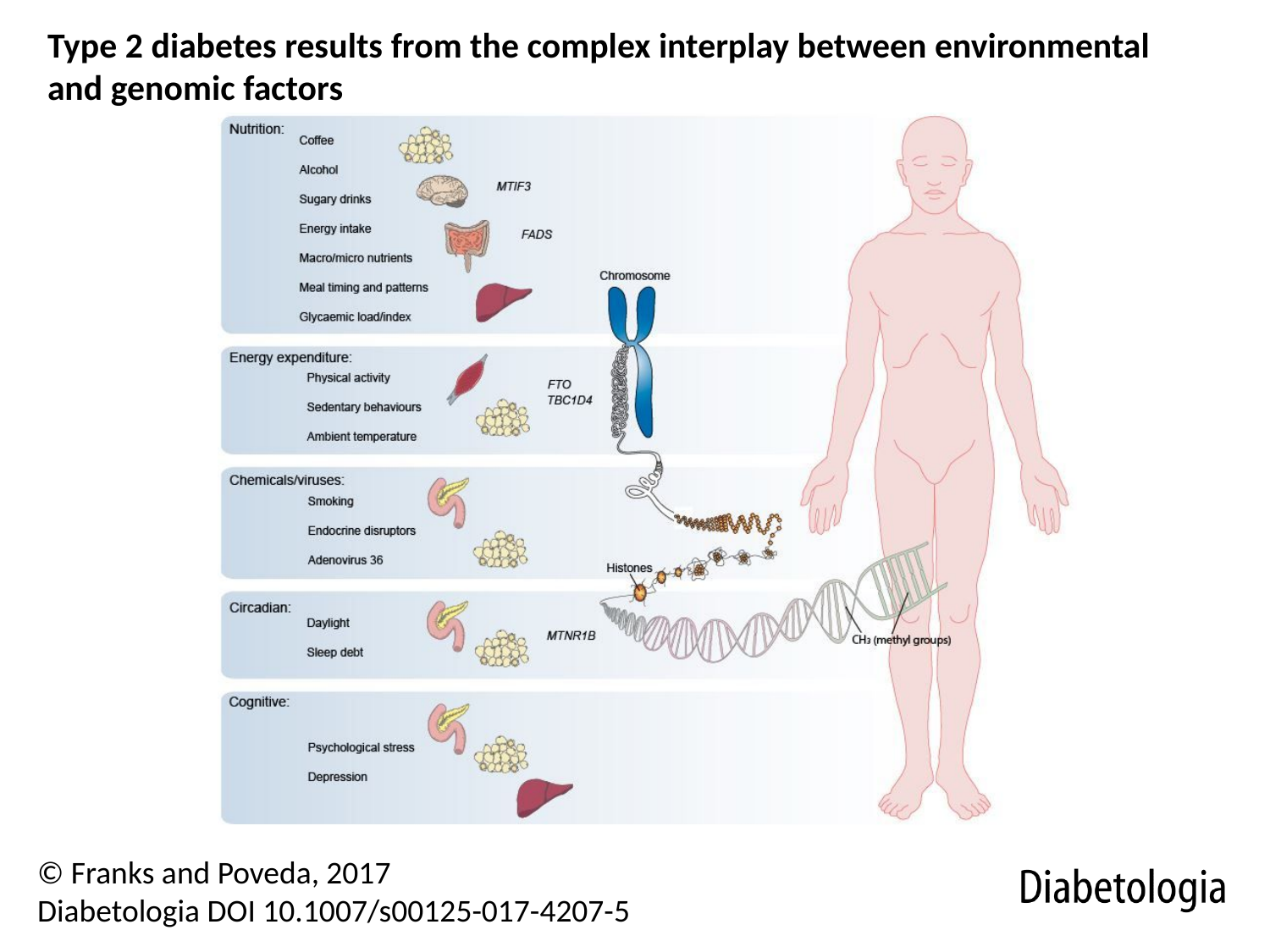

Type 2 diabetes results from the complex interplay between environmental and genomic factors
© Franks and Poveda, 2017
Diabetologia DOI 10.1007/s00125-017-4207-5

## Slide 2
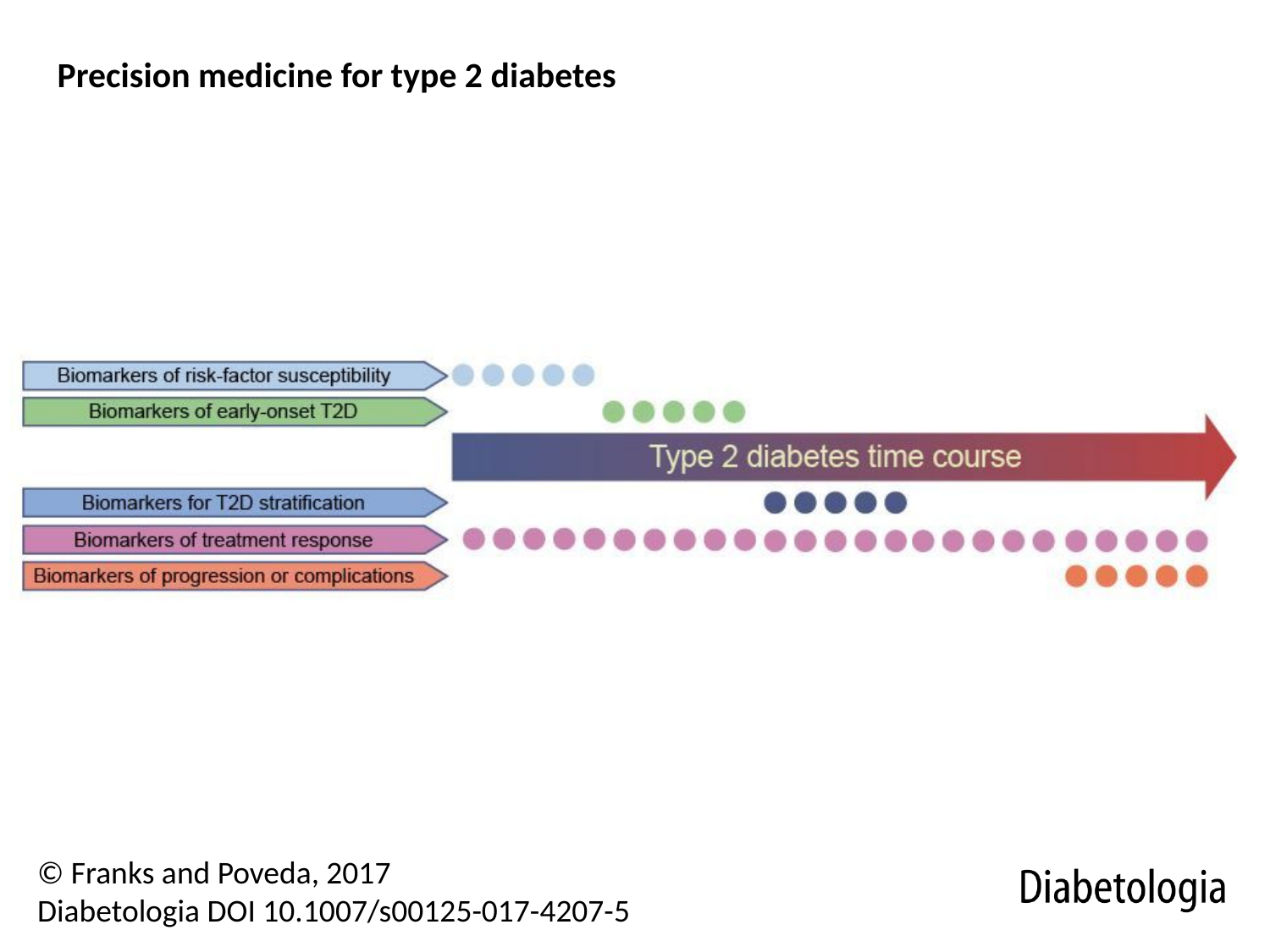

Precision medicine for type 2 diabetes
© Franks and Poveda, 2017
Diabetologia DOI 10.1007/s00125-017-4207-5

## Slide 3
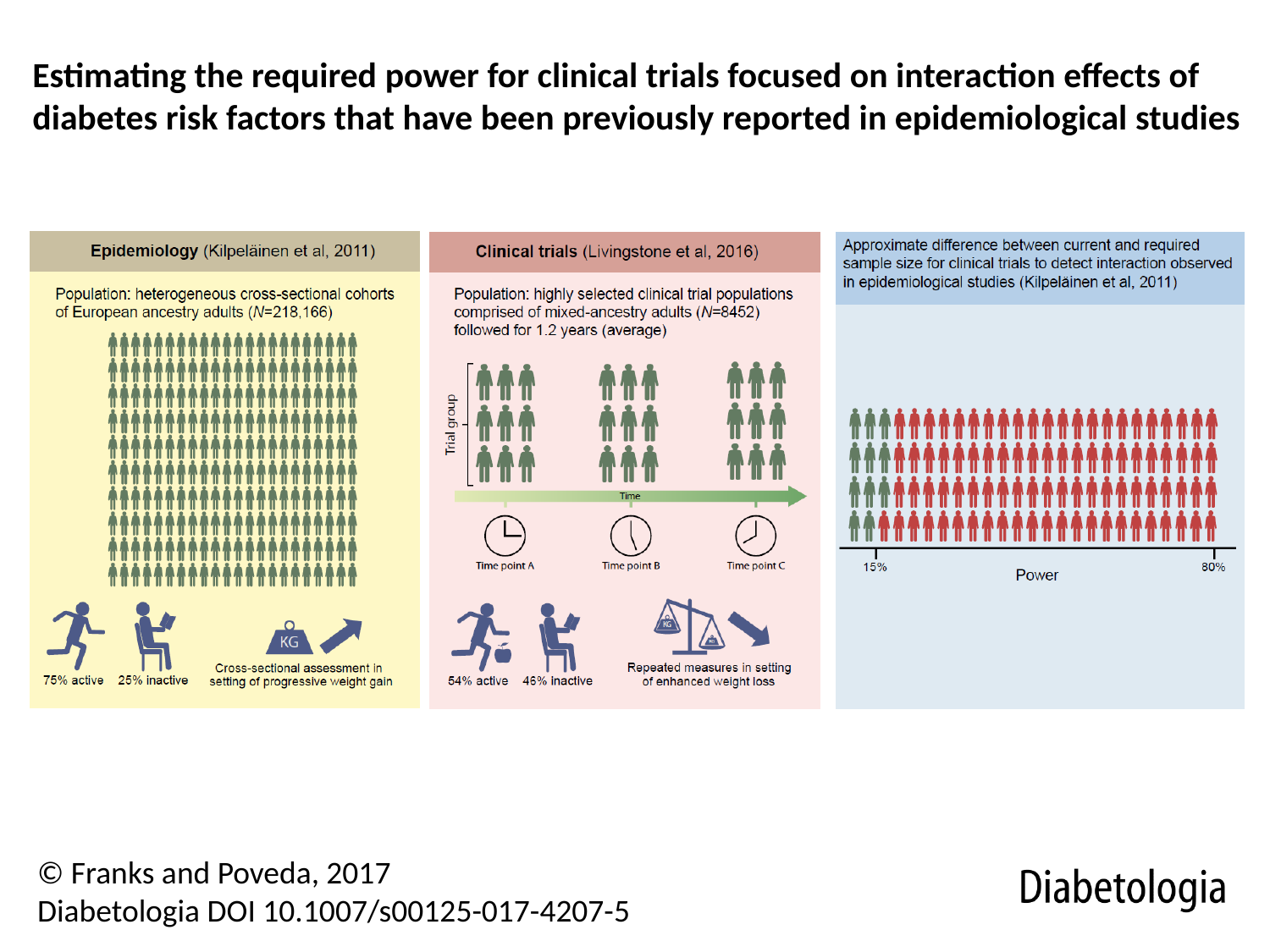

Estimating the required power for clinical trials focused on interaction effects of diabetes risk factors that have been previously reported in epidemiological studies
© Franks and Poveda, 2017
Diabetologia DOI 10.1007/s00125-017-4207-5
